# Cellular and molecular characterization of γδ T cells in peripheral blood from patients with metastases from cutaneous and uveal melanoma

**DOI:** 10.3389/fimmu.2025.1564333

**Published:** 2025-07-28

**Authors:** Tamara Alonso-Agudo, Junko Johansson, Anders Ståhlberg, Roger Olofsson Bagge

**Affiliations:** ^1^ Department of Surgery, Institute of Clinical Sciences, Sahlgrenska Academy, University of Gothenburg, Gothenburg, Sweden; ^2^ Wallenberg Centre for Molecular and Translational Medicine, University of Gothenburg, Gothenburg, Sweden; ^3^ Department of Laboratory Medicine, Institute of Biomedicine, Sahlgrenska Academy, University of Gothenburg, Gothenburg, Sweden; ^4^ Department of Clinical Genetics and Genomics, Sahlgrenska University Hospital, Gothenburg, Sweden; ^5^ Department of Surgery, Sahlgrenska University Hospital, Gothenburg, Sweden

**Keywords:** γδ T cells, TRD, TCR, cutaneous melanoma, uveal melanoma, isolated regional perfusion, ultrasensitive DNA sequencing, SiMSen-Seq

## Abstract

T cells can be divided into two major subtypes based on which chains that make up the T cell receptor (TCR): the conventional αβ T cells and the less common γδ T cells. γδ T cells are attractive targets of cancer immunotherapy due to e.g. their independence from MHC-restricted activation. Despite the successful implementation of immune checkpoint inhibitors for the treatment of metastatic melanoma, not all patients respond favorably to the treatment. In this study we characterized γδ T cells in peripheral blood from patients with cutaneous and uveal melanoma, and from age-matched healthy controls, with ultrasensitive DNA sequencing (SiMSen-Seq) of the δ-chain and flow cytometry to facilitate the introduction of γδ T cell-based treatment strategies. As a general trend, the Vδ1^+^ subpopulation was found to be more abundant in patients labeled as responders versus non-responders. Regarding clonal diversity, although a high oligoclonality was found in each individual and within each group, clonal diversity was lower in patients labeled as responders to treatment. Cutaneous melanoma patients had a larger total number of clonotypes compared to the healthy controls, and did also express higher levels of the receptor NKG2D on the surface of Vδ2^+^ cells. Overall, we could see small differences between cutaneous and uveal melanoma patients and healthy controls in regard to distribution of γδ subpopulations, with high clonal diversities and a mostly private repertoire of the δ receptor among all groups.

## Introduction

Cutaneous melanoma is a commonly occurring skin cancer, most prevalent among fair-skinned populations of European descent (1). In Sweden, melanoma is the fifth most common form of cancer, with incidence rates that have been increasing during the last decades (2). Uveal melanoma is much rarer and constitutes around only 5% of patients with melanoma, though it is the most common intraocular tumor in adults (3, 4). During recent years immune checkpoint inhibition has revolutionized the treatment for patients with cutaneous melanoma, but unfortunately with limited efficacy for patients with uveal melanoma. Therefore, there is a need for an improved understanding of the immunological milieu in patients with the disease.

T cells are one of the most utilized immune cells for immunotherapeutic treatment regimes. They can be divided into two major subtypes based on which protein chains that make up the T cell receptor (TCR): the more abundant αβ T cells, and the less studied γδ T cells. γδ T cells are established to have a non-MHC-restricted activation and can be activated instead by phosphoantigens. They home mainly to peripheral barrier tissues, although they can also be found in peripheral blood at low frequencies, and they have a predominant antitumor effect (5–7). However, in rare events, an inflammatory milieu can polarize γδ T cells into IL-17-producing cells with a protumor effect (6). γδ T cells are attractive targets of cancer immunotherapy due to e.g. their independence from MHC-restricted activation (6, 7). Indeed, studies have shown that they might be efficient targets of immune checkpoint inhibitors in the treatment of melanoma (8–10), highlighting the need for an expanded and deeper characterization of the γδ T cell immune repertoire.

The γδ T cell receptor is encoded by the gamma and delta loci (*TRG* and *TRD*, respectively). The *TRD* locus contains eight variant alleles (*TRDV*; 5 of them are shared with *TCR* alpha), whose usage defines the different human γδ T cell subsets, three diversity alleles (*TRDD*), four joining alleles (*TRDJ*), and one constant (*TRDC*) region. Somatic recombination of these *TRD* alleles, result in the complementary-determining region 3 (CDR3) (11), and when γδ T cells are activated, the somatic recombination in their antigen receptors passes on to the expanded clones. Thus, the immune repertoire can be studied through their CDR3 (11).

As every T cell has a unique CDR3 sequence, immune sequencing calls for an accurate base-calling technique and little to no amplification bias. In addition, γδ T cells in peripheral blood are sparse (6), thus needing a sensitive method, and quantification of the number of cells is key for the study of clonal expansion. The ultrasensitive immune repertoire sequencing approach based on SiMSen-Seq constitutes a good solution to the existing challenges (12). This targeted sequencing method utilizes unique molecular identifiers (UMIs) that are bioinformatically used for creating consensus reads, effectively obtaining close to error-free sequence data, and minimal biases (13).

Here, we have combined the powerful sensitivity of novel SiMSen-Seq technology together with traditional flow cytometric immunophenotyping in an attempt to define the immune repertoire of γδ T cells in patients with cutaneous and uveal melanoma.

## Materials and methods

### Patients and purification of PBMCs

Peripheral blood samples were obtained from 14 patients with cutaneous melanoma in-transit metastases and 14 patients with uveal melanoma liver metastases. Sampling occurred between 2014 and 2021. At the time of sampling, all patients had not received any systemic treatment for their disease. All patients were scheduled for a locoregional cancer treatment with isolated limb perfusion (ILP) or isolated hepatic perfusion (IHP). Both methods aim to deliver a very high dose of the alkylating chemotherapeutic agent melphalan to either an extremity (ILP) or to the liver (IHP), without causing any systemic side effects. In short, the limb or the liver is surgically isolated and connected to a heart-lung machine and for approximately 1 hour the organ or limb is then perfused with melphalan, after this all chemotherapy is rinsed away and the anatomy is restored.

Three months after the procedure the treatment response was evaluated according to RECIST 1.1 criteria as complete response (CR, defined as disappearance of all lesions), partial response (PR, decrease of more than 30% of total tumor burden), progressive disease (PD, an increase of more than 25% in existing lesions or the appearance of new lesions) or stable disease (SD, where none of the criteria for CR, PR or PD were met) (14). In this study CR and PR were grouped together as responders, while PD and SD were collectively called non-responders.

Peripheral blood samples were also obtained from 13 age-matched healthy controls during 2022. Information about all controls can be found in **Supplementary Table 1**. Consent was given by all participants, and the study has been approved by the Regional Ethical Review Board in Gothenburg, Sweden (995-16).

Peripheral blood mononuclear cells (PBMCs) from both patients and healthy donors were obtained from BD Vacutainer^®^ CPT™ cell preparation tubes (BD Biosciences, #362782) according to the manufacturer’s protocol. Cells were resuspended and frozen in Recovery™ Cell Culture Freezing Medium (ThermoFisher Scientific, #12648010) followed by cryopreservation in liquid nitrogen until further usage.

### Enrichment of γδ T cells and subsequent DNA extraction

γδ T cells were enriched from thawed PBMCs with magnetic cell separation. Enrichment was performed with a TCRγ/δ^+^ T Cell Isolation Kit (#130-092-892) and LS columns on MidiMACS™ and QuadroMACS™ Separators, all from Miltenyi Biotec and according to the manufacturer’s protocol. DNA from enriched γδ T cells were purified with QIAamp DNA Blood Mini Kit (Qiagen, #51104) and concentrated with Vivacon^®^500 30kDa centrifugal units (VWR, #518-0002). The concentration of DNA was quantified with Qubit™ 1X dsDNA HS Assay Kit (ThermoFisher Scientific, #Q32851) on a Qubit^®^ 3.0 Fluorometer.

### Library construction and sequencing

Primers targeting the T cell receptor delta (*TRD*) repertoire, previously designed by Johansson et al. (12), were used for library construction. Forward primers targeted downstream all 8 variable (*TRDV*) genes, while reverse primers targeted downstream all 4 joining (*TRDJ*) genes, allowing amplification of the CDR3 region.

Triplicates of 20 ng of DNA of enriched γδ T cells were used per patient for library construction, making a total of 60 ng of DNA per patient.

Barcoding of DNA was performed in a reaction containing 0.05 U Platinum™ SuperFi™ DNA Polymerase, 1x SuperFi Buffer (both ThermoFisher Scientific, #12351010), 0.2 nM dNTP Mix (Thermo Fisher Scientific, #R0191), 0.5M L-carnitine inner salt (Sigma-Aldrich, #C0158), 40 nM of each barcode primer (Ultramer™ DNA Oligo standard desalting purified, Integrated DNA Technologies), target DNA, and Ultrapure™ DNase/RNase-Free Distilled Water (Thermo Fisher Scientific, #10977035) until reaching a total volume of 10 μl. The following temperature program was used on a T100 Thermal cycler (Bio-Rad Laboratories): 98 ˚C for 30 sec, 3 cycles of amplification (98 ˚C for 10 sec, 62 ˚C for 6 min, 72 ˚C for 30 sec, ramping rates 4 ˚C/sec), followed by 65 ˚C for 15 minutes and 95 ˚C for 15 min. Before the 15 minutes incubation at 65 ˚C, 20 μl of 45 ng/μl *Streptomyces griseus* protease (Sigma-Aldrich, #P5147) dissolved into RNase-free TE buffer (pH 8.0, Thermo Fisher Scientific, #AM9858) was added to each reaction to reduce non-specific product formation by degrading and inactivating the DNA polymerase.

Adapter amplification was performed in a second PCR with a total reaction volume of 40 μl, containing 1x Q5^®^ Hot Start High-Fidelity Master Mix (New England BioLabs, #M0494), 400 nM of each Illumina Adapter index primer (desalted, Integrated DNA Technologies), 10 μl of the barcoded and diluted PCR product and Ultrapure™ DNase/RNase-Free Distilled Water (Thermo Fisher Scientific, #10977035). The following temperature program was used on a T100 Thermal cycler: 98 ˚C for 3 min, 28 to 30 cycles of amplification (98 ˚C for 10 sec, 80 ˚C for 1 sec, 72 ˚C for 30 sec, ramping rates 0.2 ˚C/sec).

Automated parallel capillary electrophoresis was used for quality control and quantification of libraries with the HS NGS Fragment kit (Agilent Technologies, #DNF-474) on a Fragment Analyzer, using the PROsize software version 3.0 (Agilent Technologies). All according to the manufacturer instructions. Our library pool was then purified using a 2% agarose, 100-600bp DNA gel cassette (Sage Science, #CEF2010) on a Pippin Prep (Sage Science) with a broad range program from 210 to 400 bp. Final quantification of the purified library pool was performed with NEBNext Library Quant Kit (New England Biolabs, #E7630) according to manufacturer’s instructions, using a CFX384 Touch Real-Time PCR Detection System instrument (Bio-Rad Laboratories).

Paired-end sequencing was performed on a MiniSeq (Illumina), using a mid-output reagent kit (300-cycles) with 10% added PhiX control v3 (Illumina, #FC-420–1004 and #FC-110-3001) and 1.8 pM library.

### Sequencing data analysis

Sequencing data were processed for de-multiplexing and error-correction using the molecular identifier guided error correction (MIGEC) software (15), version 1.2.9, with paired-end reads, overlap-max-offset set to 40 for the Checkout routine, and force-overseq set to 2 during assembly. Final output data after filtering out non-functional (ie.: non-coding) clonotypes resulted in so-called productive clonotypes with their respective abundance, which were analyzed using immunarch R programming package, version 0.6.9 (16), and treemap plots were created using treemapify R programming package, version 2.5.5.

All clonotypes with a CDR3 amino acid sequence starting by “CALPI” (CALPI-like clonotypes) were removed from the analysis, as they were an artifact with origin in the TRDJ primers (**Supplementary Table 2**).

### Stimulation of γδ T cells for cytokine measurement

Unstained PBMCs were seeded overnight in RPMI 1640 medium (Sigma-Aldrich, #R7388-500ML) containing 10% heat-inactivated fetal bovine serum (Sigma-Aldrich, #F7524) and 1% penicillin/streptomycin (Cytiva, #SV30010). PBMCs were counted and seeded at a concentration of 1·10^6^ cells/ml in round-bottomed 96-wells plates in complete medium with 25 ng/ml phorbol 12-myristate 13-acetate (PMA, Sigma-Aldrich, #P1585-1MG), 1 μg/ml ionomycin calcium salt (Sigma-Aldrich, #I0634-1MG) and 1 μl/ml BD GolgiPlug™ containing brefeldin A (BD Biosciences, #555029), 0.2 ml/well. They were incubated at 37°C with 5% CO_2_ for 4 hours, followed by staining with viability dye and antibodies for flow cytometry analysis.

### Flow cytometry

Cells were stained with extracellular antibodies in phosphate buffered saline (PBS) with 0.5% BSA and 0.1% EDTA. Stainings for intracellular antibodies were done in permeabilization buffer following fixation and permeabilization of the cells (Invitrogen™ eBioscience™ Foxp3/Transcription Factor Staining Buffer Set, Fisher Scientific, #11500597). A LIVE/DEAD™ Fixable Far Red Dead Cell Stain Kit (ThermoFisher Scientific, #L34974) was used for viability analyses and added prior to the antibody stainings. A list of all conjugated antibodies can be found in **Supplementary Table 3**. Gatings strategies are shown in **Supplementary Figures 1**, **2**. Flow cytometry analyses were conducted on a BD LSRFortessa™ instrument (BD Biosciences) running BD FACSDiva Software version 9.0.1 (BD Biosciences). Subsequent data analysis was performed in Kaluza Analysis Software version 2.3 (Beckman Coulter).

### Statistics

Statistical analyses for flow cytometry data were performed in GraphPad Prism 10 (GraphPad Software) with all tests being non-parametric and unpaired Mann-Whitney tests. GraphPad Prism 10 were also used for some of the analyses of the sequencing data, wherein non-parametric and unpaired Kruskal-Wallis tests were performed followed by Dunn’s test with correction for multiple comparisons.

The rest of the statistical analysis of the sequencing data was performed with immunarch R program, using Kruskal-Wallis tests and p-value adjusting with Holm method.

## Results

### Characterization of γδ T cells in melanoma patients and healthy controls

**Figure 1:**
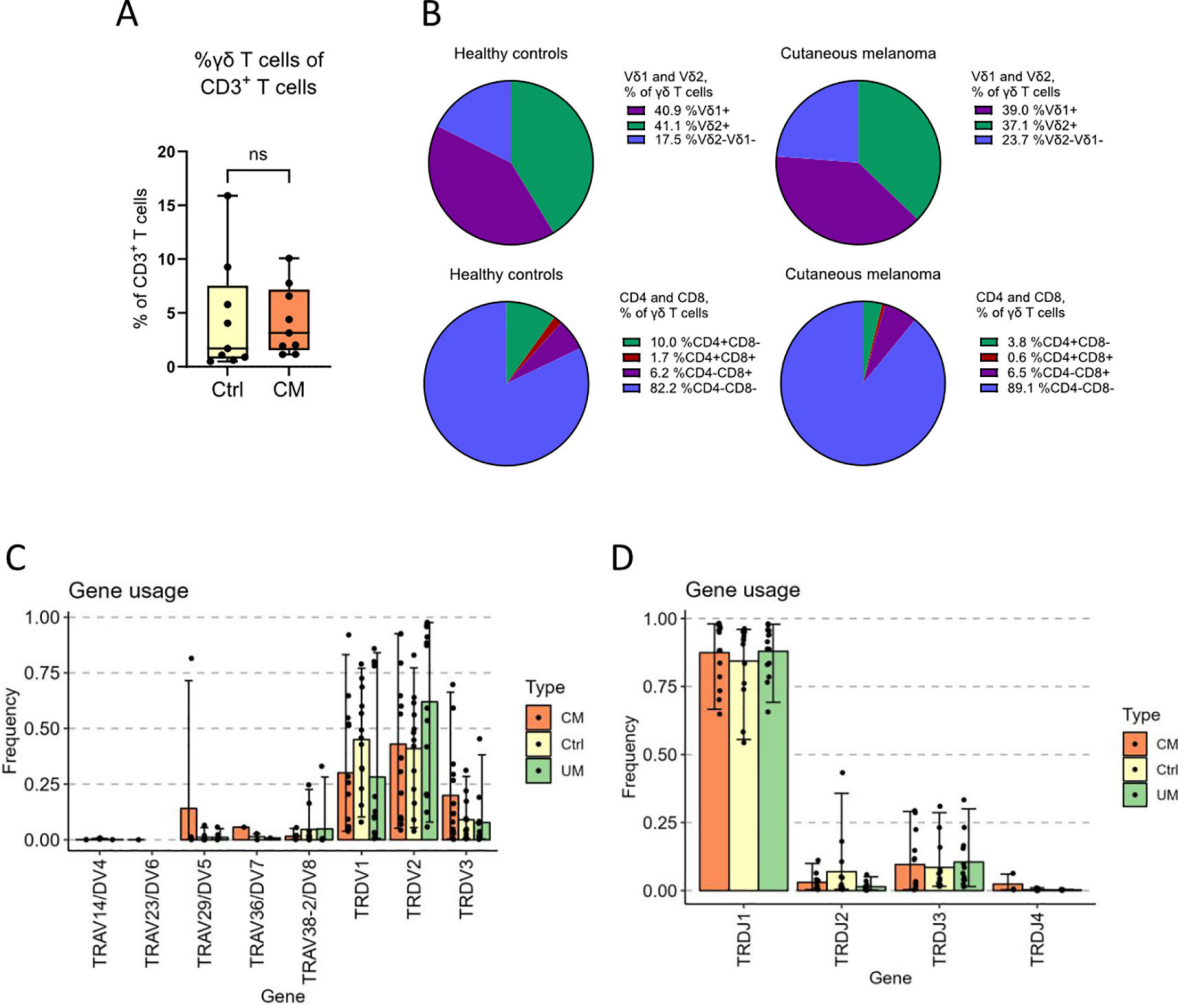
γδ T cell subsets and gene usage analysis. γδ T cells from cutaneous melanoma patients and healthy controls were characterized by flow cytometry. **(A)** The percentage of total γδ T cells among all living CD3^+^ T cells in peripheral blood and **(B)** the distribution of Vδ1, Vδ2, CD4 and CD8 among those γδ T cells (n_CM_=9, n_Ctrl_=9, unpaired Mann-Whitney test, ns, not significant). **(C)** The frequency of V-genes and **(D)** J-genes used by all the clonotypes of a group, weighed by the abundance of said clonotypes. Ctrl, Control; CM, Cutaneous melanoma; UM, Uveal melanoma.

Flow cytometry, as well as sequencing of the *TRD* gene, were used for basic characterization of γδ T cells from patients with melanoma in-transit metastasis and from age-matched healthy controls, while γδ T cells from uveal melanoma patients with liver metastasis were only subjected to sequencing. Flow cytometry results showed no difference in the percentage of living γδ T cells between patients with cutaneous melanoma and healthy controls (**Figure 1A**). Surprisingly, both groups presented very low values, given that the samples had been enriched for γδ T cells. Both cutaneous melanoma patients and healthy controls showed similar percentages for Vδ1^+^ and Vδ2^+^ T cells by flow cytometry (**Figure 1B**). Flow cytometry data also showed that cutaneous melanoma patients presented a trend towards a higher percentage of Vδ1^-^Vδ2^-^ T cells than healthy controls, and for both groups the vast majority of γδ T cells were CD4^-^CD8^-^ (**Figure 1B**). Sequencing results showed that gene usage of *TRDV* had very high variation among individuals, although Vδ1^+^ and Vδ2^+^ were the most abundant (**Figure 1C**; **Supplementary Table 4**). No statistically significant differences in gene usage were found among groups (control, cutaneous melanoma and uveal melanoma). However, as a general trend, the *Vδ1* gene was more abundant in the healthy control individuals, while the *Vδ2* gene was more abundant in uveal melanoma patients. For the *TRDJ* gene usage, the *TRDJ1* population comprised the vast majority of γδ T cells in all three groups (**Figure 1D**, **Supplementary Table 4**), which was expected given the *Vδ2* gene bias for *TRDJ1* (11, 12).

### γδ T cell diversity and clonality distribution in melanoma patients and healthy controls

**Figure 2:**
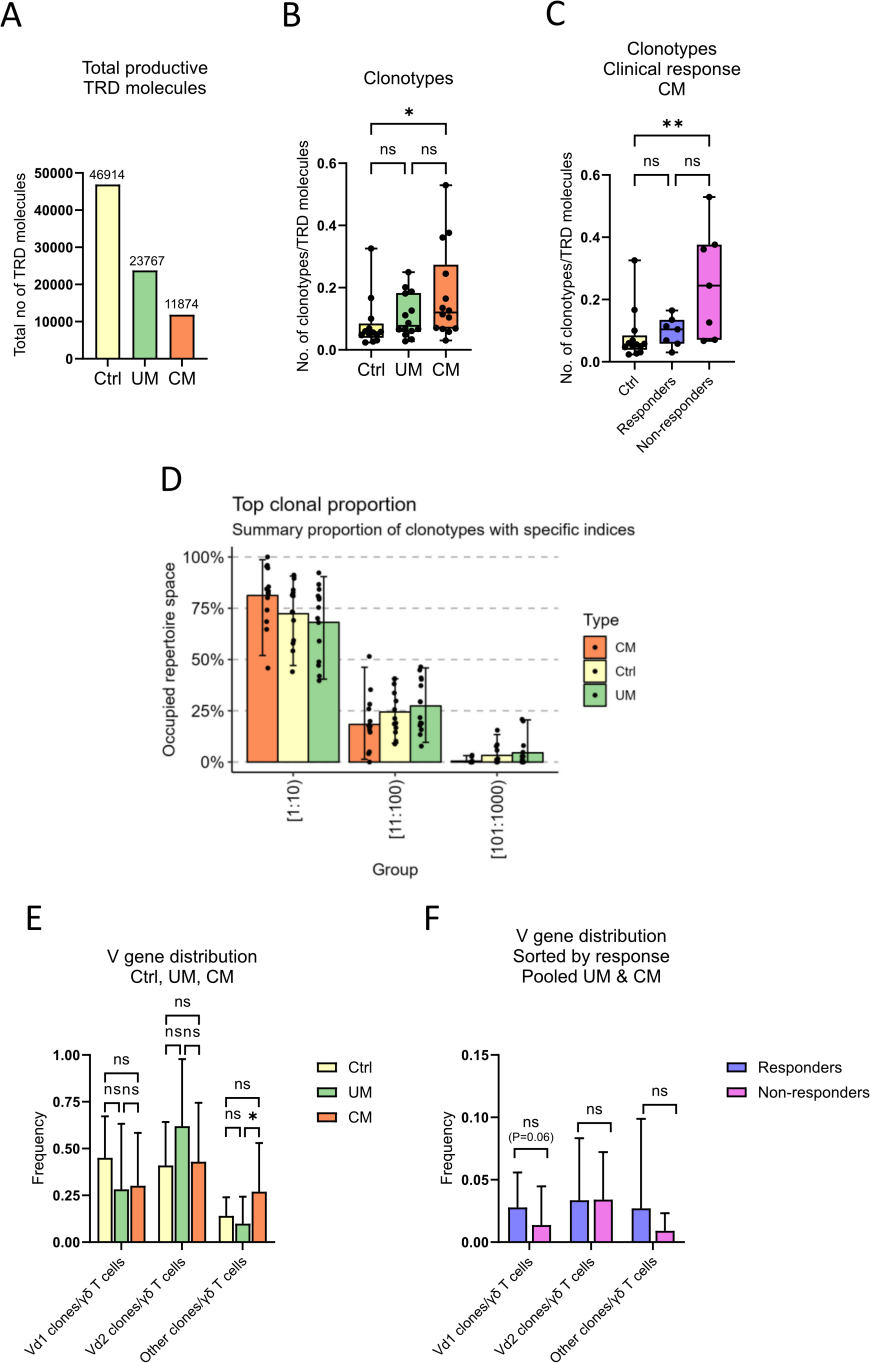
γδ T cell diversity and clonality distribution. Sequencing data of γδ T cells was used to analyze their between and in-group diversity and clonality distribution. **(A)** Sum of productive TRD molecules in each group. Number of clonotypes per total productive TRD molecules ratio, **(B)** compared by groups and **(C)** compared by clinical response (n_Ctrl_=13, n_CM_=14, n_UM_=14, n_Responders_=7, n_Non-responders_=7, unpaired Kruskal-Wallis test followed by Dunn’s multiple comparisons test, **P<0.01, *P<0.05, ns, not significant). **(D)** Percentage of total repertoire space occupied by the 1000 most abundant clonotypes in order of proportion (top 10, 11^th^ to 100^th^, and 101^st^ to 1000^th^ most abundant clonotypes), compared by groups. **(E)** Relative abundance of clonotypes by *TRDV* gene usage per group (n_Ctrl_=13, n_CM_=14, n_UM_=14, unpaired Kruskal-Wallis test followed by Dunn’s multiple comparisons test, *P<0.05, ns, not significant). **(F)** Relative abundance of clonotypes sorted by responders or non-responders in pooled melanoma samples, control samples excluded (n_Responders_=13, n_Non-responders_=15, un-paired Mann-Whitney test followed by Dunn’s multiple comparisons test, ns, not significant). Ctrl, Control; CM, Cutaneous melanoma; UM, Uveal melanoma.

The *TRD* gene of γδ T cells of cutaneous melanoma, uveal melanoma patients, and of healthy controls, was sequenced and its clonality analyzed. Sample diversity was estimated using clonotypes per total *TRD* molecules ratio. It is notable that fewer productive *TRD* molecules were detected in samples from cutaneous melanoma patients than in uveal melanoma and healthy control samples (**Figure 2A**; 11874, 23767 and 46914 total *TRD* molecules detected, respectively), despite using the same amount of DNA. The number of clonotypes per total number of productive *TRD* molecules detected in each group were compared, and it was found that the ratio was significantly lower for the control group than for cutaneous melanoma patients (**Figure 2B**), suggesting a higher diversity in cutaneous melanoma patients. Further investigation of cutaneous melanoma patient sample diversity revealed a significant difference in clonotype to *TRD* molecules ratio between healthy controls and non-responders (**Figure 2C**), indicating that patients who do not respond to the treatment have a highly diverse *TRD* repertoire.

Visualization of clonality distribution showed that the mean occupied repertoire space of the top ten clonotypes in the three groups was >65%, demonstrating a high oligoclonality of samples regardless of groups (**Figure 2D**). Relative abundance of clonotypes by *TRDV* gene usage was calculated using the ratio between number of certain *TRDV* population clones per total number of productive *TRD* molecules detected per sample. There was a higher relative abundance of non-Vδ1/2 clonotypes for patients with cutaneous melanoma, compared to healthy control individuals and uveal melanoma patients (**Figure 2E**).

In order to analyze differences in relative abundance of *TRDV* populations between patients that responded to the treatment (complete or partial response) and non-responders, data from both cutaneous and uveal melanoma patients were analyzed together. **Figure 2F** shows a trend of higher Vδ1^+^ relative abundance in responders than in non-responders (non-significant, p = 0.06).

### Immune repertoire sequencing of *TRD* gene in melanoma patients and healthy controls

**Figure 3:**
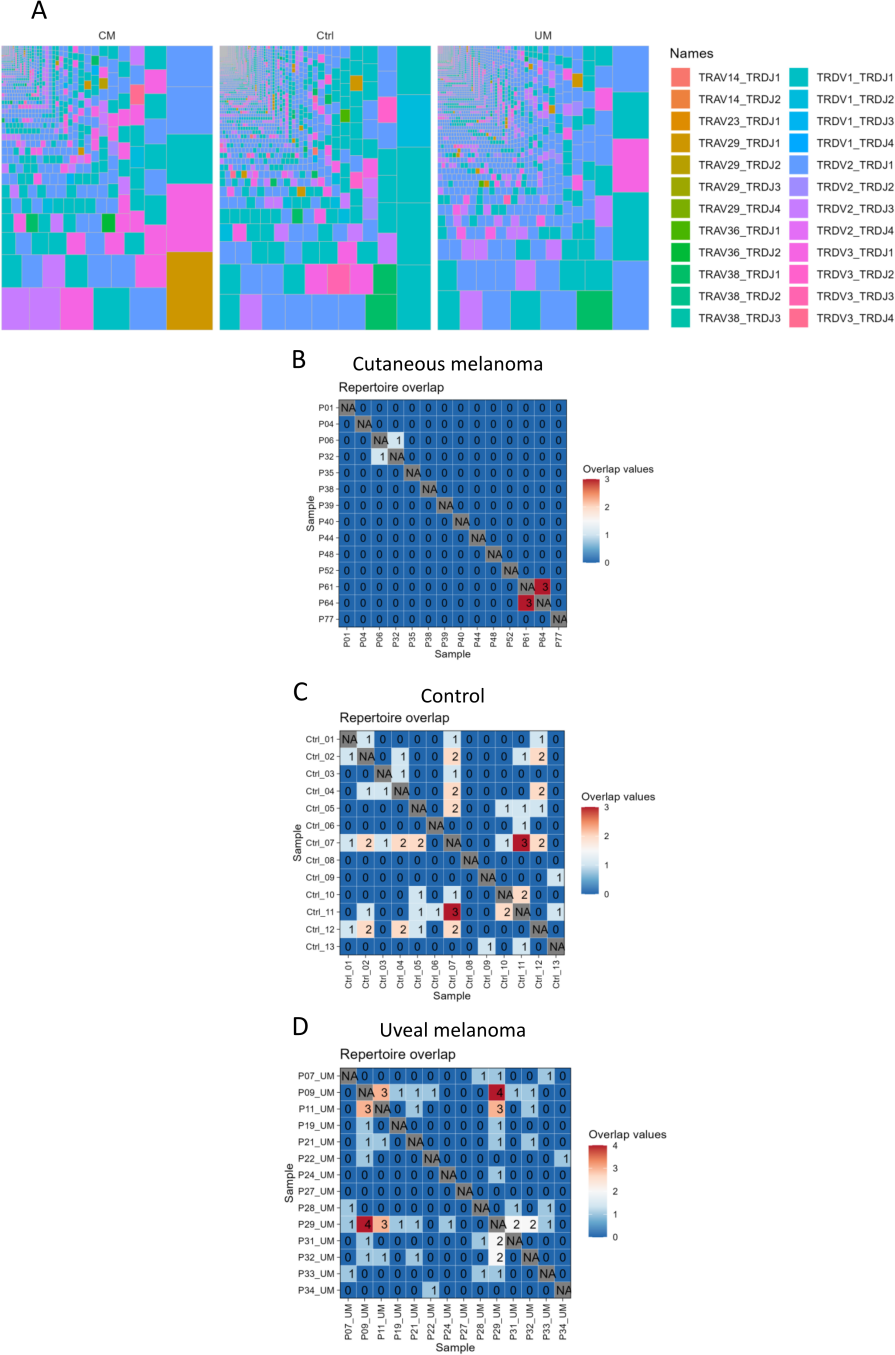
γδ T cell repertoire sequencing. Repertoire of γδ T cells was **(A)** condensed into treemaps of all clonotypes within each group. Colors show the combination of *TRDV* and *TRDJ* genes per clonotype. Also, the repertoire of each sample was tested **(B–D)** for repetition of its individual clonotypes in other samples of the same group, obtaining a maximum overlap value of 4 clonotypes shared between two uveal melanoma samples.

Sequencing of the *TRD* gene was performed for repertoire characterization of γδ T cells from blood samples in cutaneous and uveal melanoma patients, as well as age-matched healthy controls. Repertoire diversity of *TRD* clonotypes showed a similar distribution between groups, with highly oligoclonal individuals (**Supplementary Figures 3B–D**) but without clear dominating clonotypes within each group (**Figure 3**, **Supplementary Figure 3A**). There was, in fact, little repertoire overlap between samples within each group (**Figures 3B–D**). The majority of samples shared no clonotypes (zero repertoire overlap value), and the maximum overlap values were 3 for cutaneous melanoma patients and healthy controls, and 4 for uveal melanoma patients, meaning that samples showed mostly a private TRD repertoire.

### Functionality of γδ T cells in melanoma patients and in healthy controls

**Figure 4:**
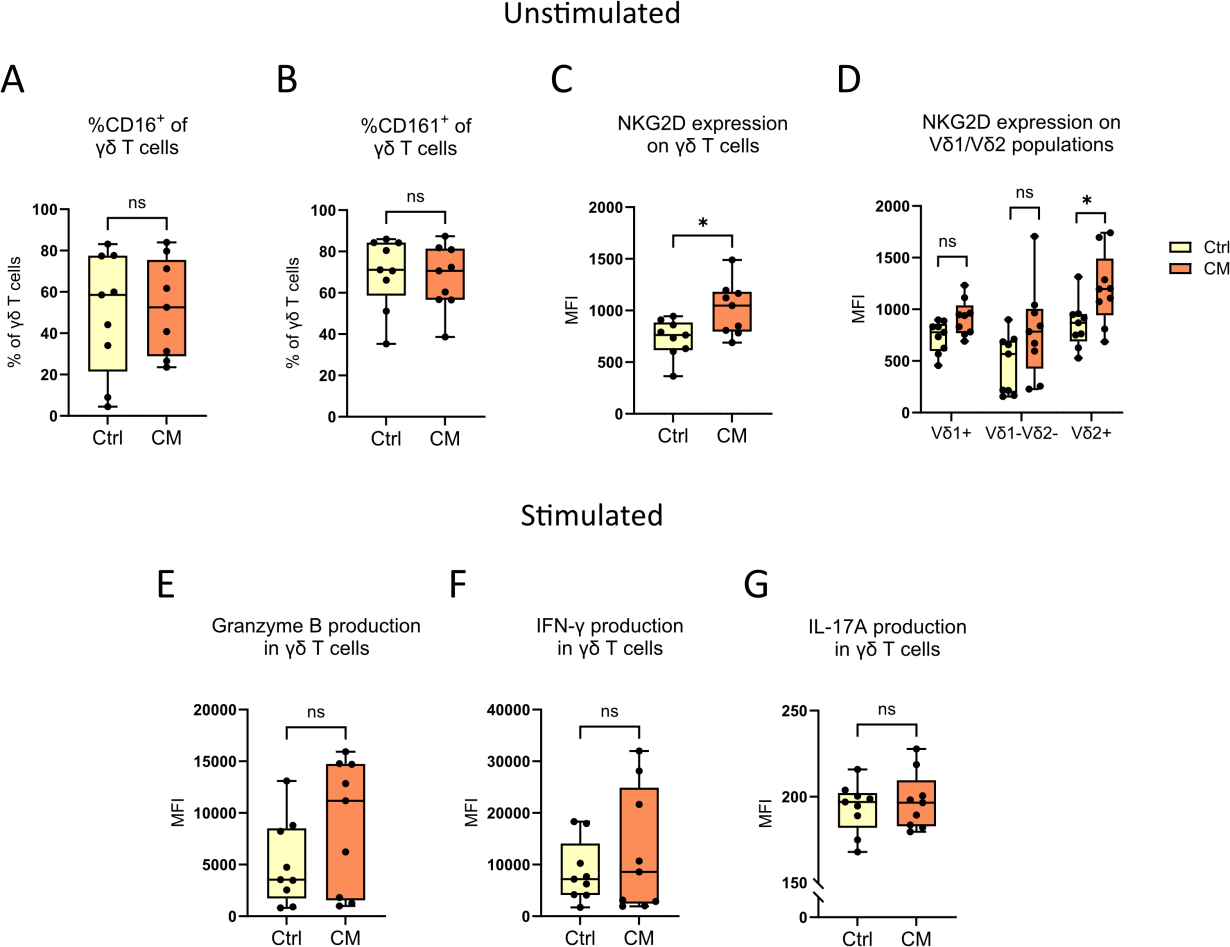
γδ T cell functionality studies. The extracellular expression of **(A)** CD16, **(B)** CD161 and **(C, D)** NKG2D on peripheral γδ T cells from cutaneous melanoma patients and healthy controls were characterized by flow cytometry. γδ T cells from the same samples were also stimulated with PMA and ionomycin for 4 hours before subsequent flow cytometry analysis of the intracellular expression of **(E)** granzyme B, **(F)** IFN-γ and **(G)** IL-17A. n_CM_=9, n_Ctrl_=9, unpaired Mann-Whitney test, *P<0.05, ns, not significant. MFI, Median fluorescence intensity; Ctrl, Control; CM, Cutaneous melanoma.

The expression of the extracellular receptors CD16, CD161 and NKG2D on γδ T cells from both the patients with cutaneous melanoma and from healthy controls was analyzed with flow cytometry. NKG2D, CD16 and CD161 are commonly found on natural killer cells and on IL-17-producing T cells (17–19) and were here utilized as an indirect way of measuring potential cytotoxicity. There were no differences in the percentages of CD16^+^ and CD161^+^ γδ T cells between patients and healthy controls (**Figures 4A, B**), but γδ T cells from patients had higher levels of NKG2D (**Figure 4C**). Further investigation showed that the observed difference in NKG2D was found only on Vδ2^+^ γδ T cells (**Figure 4D**).

To investigate the functionality of γδ T cells in more detail, PBMCs from cutaneous melanoma patients and healthy controls were stimulated with two compounds commonly used to activate T cells; PMA, a protein kinase C activator, and ionomycin, a Ca^2+^ ionophore (20, 21). Following stimulation, the secretion of granzyme B, IFN-γ and IL-17A was determined. No differences were found between melanoma patients and healthy controls (**Figures 4E–G**), and a deeper analysis focused on Vδ1/Vδ2^+^ γδ T cells neither showed any differences (**Supplementary Figures 4A–C**).

## Discussion

In line with previous studies, analysis of the total level of γδ T cells by flow cytometry showed no difference between patients with cutaneous melanoma and age-matched healthy controls (8, 22–25). We also observed a very similar distribution of the Vδ1^+^ and Vδ2^+^ subsets between the melanoma patients and the controls, with none of the subsets being more prevalent than the other. This contrasts with established literature which states that peripheral blood is dominated by the Vδ2^+^ subpopulation (5, 6, 26). However, there is no clear consensus in literature of the exact ratio of the different γδ T cells subsets in peripheral blood from melanoma patients compared to healthy controls, with reports of no difference in percentages of Vδ1^+^ and Vδ2^+^ cells (23), higher Vδ2^+^/Vδ2^-^ ratio for patients (22), and lower levels of Vδ2^+^ cells in patients (8, 25, 27).

Interestingly, there seems to be a trend in literature towards a better clinical outcome with longer progression-free survival and less advanced disease for melanoma patients with increased levels of Vδ2^+^ γδ T cells (22–24). In addition, one study reported a longer overall-survival after treatment with the immune checkpoint inhibitor ipilimumab for melanoma patients with low frequencies of Vδ1^+^ and high frequencies of Vδ2^+^ γδ T cells (8).

In this study, sequencing data mainly confirmed the flow cytometry results. The *Vδ1* and *Vδ2* genes did not significantly differ between patients (both cutaneous and uveal melanoma) and healthy controls (8, 25, 27). Although, the *Vδ2* gene did follow a trend of higher abundance in metastatic uveal melanoma patients. It is notable, however, that we also found a reduced number of productive *TRD* molecules in melanoma patients compared to that found in healthy controls via sequencing. Healthy controls had a two-fold higher amount of *TRD* molecules compared to the uveal melanoma patient group, which, in turn, had a two-fold higher amount of *TRD* compared to the cutaneous melanoma patient group. Despite these inconsistencies, after normalization we found two interesting trends, with higher *Vδ1* relative gene abundance in healthy controls, as well as in responders compared to non-responders (p = 0.06).

The literature on γδ T cells in uveal melanoma is very sparse. γδ T cells has been detected in enucleated eyes from patients with choroidal melanoma where a higher amount of Vδ1^+^ cells correlated to a longer survival (28), but to our knowledge this is the first study characterizing γδ T cells in peripheral blood from patients with uveal melanoma.

In general, there was a high clonal diversity for both healthy controls, cutaneous melanoma patients and uveal melanoma patients. Clonality was also very diverse within each separate melanoma patient, which matches previous literature (29, 30) demonstrating that tumor samples show a private immune repertoire, as well as specifically the human adult *TRD* repertoire being a mostly private one.

No discernible difference was seen between cutaneous melanoma patients and healthy controls regarding expression of the extracellular receptors CD16 and CD161. CD16 is commonly expressed on NK cells and is a receptor which binds to the fragment crystallizable region (Fc region) of IgG antibodies (18). When binding to IgG antibodies, which in turn are bound to target cells via the fragment antigen-binding region (Fab region), NK cells can kill the target cells through so called antibody-dependent cell-mediated cytotoxicity (ADCC) (31). γδ T cells are known to express CD16 as well, and can also initiate ADCC (32). The lectin receptor CD161 is expressed on NK cells and T cells (33), both on conventional αβ and on γδ T cells, and is connected to the production of IL-17A (19).

No differences could also be seen between cutaneous melanoma patients and healthy controls for the intracellular production of the apoptosis-inducing granzyme B (34) and the cytokines IFN-γ and IL-17A after stimulation with PMA and ionomycin. This is in line with a previous study where γδ T cells from peripheral blood from melanoma patients and healthy controls did not differ in their expression of the intracellular markers after the same stimulation (22). Interestingly, after stimulation with non-peptide phosphoantigens, which activate only the Vδ2^+^ subset in contrast to PMA and ionomycin which are general T cell activators (35), γδ T cells have shown to increase the expression of granzyme B and IFN-γ (22, 27). Thus, the choice of stimulating agent, in our case PMA and ionomycin, might explain why we could not observe any production of effector molecules or cytokines after *in vitro* stimulation.

In line with previous literature, the melanoma patients in our study had higher levels of the NKG2D receptor (22), which was driven by an elevated expression on Vδ2^+^ γδ T cells. NKG2D is found on both NK cells and T cells and binds ligands typically expressed on stressed and transformed cells, e.g. the proteins MICA/B (MHC class I polypeptide–related sequence A/B) which can be upregulated on the surface of cancer cells (17). It has been shown that binding of ligands to NKG2D causes activation in certain γδ T cell subpopulations (17).

The percentage of γδ T cells in both cutaneous melanoma patients and healthy controls were unexpectedly low after enrichment. γδ T cells have been shown to constitute up to 10% of all T cells in peripheral blood in healthy human individuals (29, 36–38), and even after enrichment the highest percentage of γδ T cells among all leukocytes were 7.3 for melanoma patients and 9 for controls in this study. This discrepancy might be due to technical issues with the enrichment protocol. Due to the limited availability of samples and predicted low amount of γδ T cells in peripheral blood, the purity and yield of each sample after enrichment was not measured. Though it is worth noting that the bioinformatic pipeline filters out all data not related to TRD so there are no confounding effects from other cell types, and it should not overly affect the flow cytometry analysis since the majority of the data is gated on events within the total γδ T cell population.

Another restraint in this study is the relatively low sample number, restricting the power of the statistical analyses. The availability of samples from patients undergoing isolated limb perfusion and isolated hepatic perfusion are limited, especially for uveal melanoma patients, since these procedures are rare treatment strategies. Thus, it was decided to prioritize the sequencing over the flow cytometry, leading to the sequencing experiments containing samples from both cutaneous and uveal melanoma patients, while the flow cytometric analyses only contained samples from cutaneous melanoma. Demographical and clinical variables should also be taken into consideration when analyzing the results. This includes for example the presence of latent viral infections. It has been shown that infections with cytomegalovirus (CMV) lead to a skewed ratio towards more Vδ2^-^ γδ T cells (39–41), making CMV serostatus an important factor to take into consideration. Unfortunately, that kind of information is not available. Since the median age of all donors in this study is above 65 years and the CMV seroprevalence in Europe for people above the age of 60 years is 65-98% (42), the probability is high that the majority of the donors are seropositive.

To conclude, this study observes small differences between cutaneous and uveal melanoma patients and healthy controls in regard to distribution of various γδ populations, with high clonal diversities among all groups. Further studies are needed to elucidate the nature of γδ T cells in melanoma patients in order to potentially facilitate the introduction of γδ T cells as a target for immunotherapy. This is especially true for uveal melanoma where effective immunotherapeutic treatments are still lacking and the literature on γδ T cells is sparse. The non-MHC-restricted activation of γδ T cells makes them interesting targets in uveal melanoma treatment regimes. Similarly to other types of cancers, uveal melanoma tumors may downregulate the expression of MHC class I molecules (42–44), which are needed for eradication by conventional αβ T cells. However, in contrast to e.g. cutaneous melanoma, downregulation of MHC class I have shown to correlate to longer survival for patients with uveal melanoma (42, 43, 45), indicating a possibility to introduce non-MHC-restricted treatment strategies.

## Data Availability

The datasets presented in this article are not readily available because the ethical approval for this study does not allow raw sequencing data to be uploaded into a data repository. Requests to access the datasets should be directed to corresponding author.

## References

[1] Arnold M Singh D Laversanne M Vignat J Vaccarella S Meheus F. Global burden of cutaneous melanoma in 2020 and projections to 2040. JAMA Dermatol. (2022) 158:495–503. doi: 10.1001/jamadermatol.2022.0160, PMID: 35353115 PMC8968696

[2] Socialstyrelsen Cancerfonden. Cancer i siffror 2023. (2023).

[3] Folberg R. The eye. In: Robbins SL Cotran RS Perkins JA Abbas AK Aster JC Kumar V, editors. Robbins and Cotran pathologic basis of disease, Ninth edition. Saunders, an imprint of Elsevier Philadelphia, PA, Philadelphia, PA (2015). p. 1319–45.

[4] Chang AE Karnell LH Menck HR. The National Cancer Data Base report on cutaneous and noncutaneous melanoma: a summary of 84,836 cases from the past decade. The American College of Surgeons Commission on Cancer and the American Cancer Society. Cancer. (1998) 83:1664–78. doi: 10.1002/(SICI)1097-0142(19981015)83:8<1664::AID-CNCR23>3.0.CO;2-G, PMID: 9781962

[5] Ribot JC Lopes N Silva-Santos B. gammadelta T cells in tissue physiology and surveillance. Nat Rev Immunol. (2021) 21:221–32. doi: 10.1038/s41577-020-00452-4, PMID: 33057185

[6] Silva-Santos B Serre K Norell H. gammadelta T cells in cancer. Nat Rev Immunol. (2015) 15:683–91. doi: 10.1038/nri3904, PMID: 26449179

[7] Park JH Lee HK. Function of gammadelta T cells in tumor immunology and their application to cancer therapy. Exp Mol Med. (2021) 53:318–27. doi: 10.1038/s12276-021-00576-0, PMID: 33707742 PMC8080836

[8] Wistuba-Hamprecht K Martens A Haehnel K Geukes Foppen M Yuan J Postow MA. Proportions of blood-borne Vdelta1+ and Vdelta2+ T-cells are associated with overall survival of melanoma patients treated with ipilimumab. Eur J Cancer. (2016) 64:116–26. doi: 10.1016/j.ejca.2016.06.001, PMID: 27400322 PMC5201188

[9] Di Simone M Corsale AM Toia F Shekarkar Azgomi M Di Stefano AB Lo Presti E. Tumor-infiltrating gammadelta T cells as targets of immune checkpoint blockade in melanoma. J Leukoc Biol. (2024) 115:760–70. doi: 10.1093/jleuko/qiae023, PMID: 38324004

[10] Davies D Kamdar S Woolf R Zlatareva I Iannitto ML Morton C. PD-1 defines a distinct, functional, tissue-adapted state in Vdelta1(+) T cells with implications for cancer immunotherapy. Nat Cancer. (2024) 5:420–32. doi: 10.1038/s43018-023-00690-0, PMID: 38172341 PMC10965442

[11] Fichtner AS Ravens S Prinz I. Human gammadelta TCR repertoires in health and disease. Cells. (2020) 9. doi: 10.3390/cells9040800, PMID: 32225004 PMC7226320

[12] Johansson G Kaltak M Rimniceanu C Singh AK Lycke J Malmestrom C. Ultrasensitive DNA immune repertoire sequencing using unique molecular identifiers. Clin Chem. (2020) 66:1228–37. doi: 10.1093/clinchem/hvaa159, PMID: 32814950

[13] Andersson D Kebede FT Escobar M Osterlund T Stahlberg A. Principles of digital sequencing using unique molecular identifiers. Mol Aspects Med. (2024) 96:101253. doi: 10.1016/j.mam.2024.101253, PMID: 38367531

[14] Eisenhauer EA Therasse P Bogaerts J Schwartz LH Sargent D Ford R. New response evaluation criteria in solid tumours: revised RECIST guideline (version 1.1). Eur J Cancer. (2009) 45:228–47. doi: 10.1016/j.ejca.2008.10.026, PMID: 19097774

[15] Shugay M Britanova OV Merzlyak EM Turchaninova MA Mamedov IZ Tuganbaev TR. Towards error-free profiling of immune repertoires. Nat Methods. (2014) 11:653–5. doi: 10.1038/nmeth.2960, PMID: 24793455

[16] Popov A ivan-immunomind MVolobueva Nazarov VI immunarch.bot Rumynskiy E. Immunomind/immunarch: immunarch 0.6.9 (0.6.9). (2022). doi: 10.5281/zenodo.6599744

[17] Raulet DH. Roles of the NKG2D immunoreceptor and its ligands. Nat Rev Immunol. (2003) 3:781–90. doi: 10.1038/nri1199, PMID: 14523385

[18] Anegon I Cuturi MC Trinchieri G PeRussia B. Interaction of Fc receptor (CD16) ligands induces transcription of interleukin 2 receptor (CD25) and lymphokine genes and expression of their products in human natural killer cells. J Exp Med. (1988) 167:452–72. doi: 10.1084/jem.167.2.452, PMID: 2831292 PMC2188858

[19] Maggi L Santarlasci V Capone M Peired A Frosali F Crome SQ. CD161 is a marker of all human IL-17-producing T-cell subsets and is induced by RORC. Eur J Immunol. (2010) 40:2174–81. doi: 10.1002/eji.200940257, PMID: 20486123

[20] Castagna M Takai Y Kaibuchi K Sano K Kikkawa U Nishizuka Y. Direct activation of calcium-activated, phospholipid-dependent protein kinase by tumor-promoting phorbol esters. J Biol Chem. (1982) 257:7847–51. doi: 10.1016/S0021-9258(18)34459-4, PMID: 7085651

[21] Chatila T Silverman L Miller R Geha R. Mechanisms of T cell activation by the calcium ionophore ionomycin. J Immunol. (1989) 143:1283–9. doi: 10.4049/jimmunol.143.4.1283 2545785

[22] Girard P Charles J Cluzel C Degeorges E Manches O Plumas J. The features of circulating and tumor-infiltrating gammadelta T cells in melanoma patients display critical perturbations with prognostic impact on clinical outcome. Oncoimmunology. (2019) 8:1601483. doi: 10.1080/2162402X.2019.1601483, PMID: 31413911 PMC6682366

[23] Toia F Buccheri S Anfosso A Moschella F Dieli F Meraviglia S. Skewed differentiation of circulating Vγ9Vδ2 T lymphocytes in melanoma and impact on clinical outcome. PloS One. (2016) 11:e0149570. doi: 10.1371/journal.pone.0149570, PMID: 26915072 PMC4767817

[24] Petrini I Pacini S Galimberti S Taddei MR Romanini A Petrini M. Impaired function of gamma-delta lymphocytes in melanoma patients. Eur J Clin Invest. (2011) 41:1186–94. doi: 10.1111/j.1365-2362.2011.02524.x, PMID: 22775565

[25] Wistuba-Hamprecht K Di Benedetto S Schilling B Sucker A SChadendorf D Garbe C. Phenotypic characterization and prognostic impact of circulating gammadelta and alphabeta T-cells in metastatic Malignant melanoma. Int J Cancer. (2016) 138:698–704. doi: 10.1002/ijc.29818, PMID: 26383054

[26] Raverdeau M Cunningham SP Harmon C Lynch L. gammadelta T cells in cancer: a small population of lymphocytes with big implications. Clin Transl Immunol. (2019) 8:e01080. doi: 10.1002/cti2.1080, PMID: 31624593 PMC6787154

[27] Argentati K Re F Serresi S Tucci MG Bartozzi B Bernardini G. Reduced number and impaired function of circulating gamma delta T cells in patients with cutaneous primary melanoma. J Invest Dermatol. (2003) 120:829–34. doi: 10.1046/j.1523-1747.2003.12141.x, PMID: 12713589

[28] Bialasiewicz AA Ma JX Richard G. Alpha/beta- and gamma/delta TCR(+) lymphocyte infiltration in necrotising choroidal melanomas. Br J Ophthalmol. (1999) 83:1069–73. doi: 10.1136/bjo.83.9.1069, PMID: 10460778 PMC1723174

[29] Ravens S Schultze-Florey C Raha S Sandrock I Drenker M Oberdorfer L. Human gammadelta T cells are quickly reconstituted after stem-cell transplantation and show adaptive clonal expansion in response to viral infection. Nat Immunol. (2017) 18:393–401. doi: 10.1038/ni.3686, PMID: 28218745

[30] Foord E Arruda LCM Gaballa A Klynning C Uhlin M. Characterization of ascites- and tumor-infiltrating gammadelta T cells reveals distinct repertoires and a beneficial role in ovarian cancer. Sci Transl Med. (2021) 13. doi: 10.1126/scitranslmed.abb0192, PMID: 33472952

[31] Lo Nigro C Macagno M Sangiolo D Bertolaccini L Aglietta M Merlano MC. NK-mediated antibody-dependent cell-mediated cytotoxicity in solid tumors: biological evidence and clinical perspectives. Ann Transl Med. (2019) 7:105. doi: 10.21037/atm.2019.01.42, PMID: 31019955 PMC6462666

[32] Braakman E van de Winkel JG van Krimpen BA Jansze M Bolhuis RL. CD16 on human gamma delta T lymphocytes: expression, function, and specificity for mouse IgG isotypes. Cell Immunol. (1992) 143:97–107. doi: 10.1016/0008-8749(92)90008-D, PMID: 1377991

[33] Lanier LL Chang C Phillips JH. Human NKR-P1A. A disulfide-linked homodimer of the C-type lectin superfamily expressed by a subset of NK and T lymphocytes. J Immunol. (1994) 153:2417–28. doi: 10.4049/jimmunol.153.6.2417, PMID: 8077657

[34] Afonina IS Cullen SP Martin SJ. Cytotoxic and non-cytotoxic roles of the CTL/NK protease granzyme B. Immunol Rev. (2010) 235:105–16. doi: 10.1111/j.0105-2896.2010.00908.x, PMID: 20536558

[35] Poupot M Fournie JJ. Non-peptide antigens activating human Vgamma9/Vdelta2 T lymphocytes. Immunol Lett. (2004) 95:129–38. doi: 10.1016/j.imlet.2004.06.013, PMID: 15388252

[36] Parker CM Groh V Band H Porcelli SA Morita C Fabbi M. Evidence for extrathymic changes in the T cell receptor gamma/delta repertoire. J Exp Med. (1990) 171:1597–612. doi: 10.1084/jem.171.5.1597, PMID: 2185330 PMC2187908

[37] Zhao Y Niu C Cui J. Gamma-delta (gammadelta) T cells: friend or foe in cancer development? J Transl Med. (2018) 16:3. doi: 10.1186/s12967-017-1378-2, PMID: 29316940 PMC5761189

[38] Esin S Shigematsu M Nagai S Eklund A Wigzell H Grunewald J. Different percentages of peripheral blood gamma delta + T cells in healthy individuals from different areas of the world. Scand J Immunol. (1996) 43:593–6. doi: 10.1046/j.1365-3083.1996.d01-79.x, PMID: 8633219

[39] Knight A Madrigal AJ Grace S Sivakumaran J Kottaridis P Mackinnon S. The role of Vdelta2-negative gammadelta T cells during cytomegalovirus reactivation in recipients of allogeneic stem cell transplantation. Blood. (2010) 116:2164–72. doi: 10.1182/blood-2010-01-255166, PMID: 20576814

[40] Davey MS Willcox CR Joyce SP Ladell K Kasatskaya SA McLaren JE. Clonal selection in the human Vdelta1 T cell repertoire indicates gammadelta TCR-dependent adaptive immune surveillance. Nat Commun. (2017) 8:14760. doi: 10.1038/ncomms14760, PMID: 28248310 PMC5337994

[41] Fowler K Mucha J Neumann M Lewandowski W Kaczanowska M Grys M. A systematic literature review of the global seroprevalence of cytomegalovirus: possible implications for treatment, screening, and vaccine development. BMC Public Health. (2022) 22:1659. doi: 10.1186/s12889-022-13971-7, PMID: 36050659 PMC9435408

[42] Blom DJ Luyten GP Mooy C Kerkvliet S Zwinderman AH Jager MJ. Human leukocyte antigen class I expression. Marker of poor prognosis in uveal melanoma. Invest Ophthalmol Vis Sci. (1997) 38:1865–72., PMID: 9286277

[43] Ericsson C Seregard S Bartolazzi A Levitskaya E Ferrone S Kiessling R. Association of HLA class I and class II antigen expression and mortality in uveal melanoma. Invest Ophthalmol Vis Sci. (2001) 42:2153–6., PMID: 11527924

[44] Anastassiou G Rebmann V Wagner S Bornfeld N Grosse-Wilde H. Expression of classic and nonclassic HLA class I antigens in uveal melanoma. Invest Ophthalmol Vis Sci. (2003) 44:2016–9. doi: 10.1167/iovs.02-0810, PMID: 12714638

[45] Jager MJ Hurks HM Levitskaya J Kiessling R. HLA expression in uveal melanoma: there is no rule without some exception. Hum Immunol. (2002) 63:444–51. doi: 10.1016/S0198-8859(02)00389-0, PMID: 12039519

