# Supplementary material for: Cellular and molecular characterization of γδ T cells in peripheral blood from patients with metastases from cutaneous and uveal melanoma
Source: Front Immunol. 2025 Jul 28;16:1564333. doi: 10.3389/fimmu.2025.1564333 (PMC12336189; doi:10.3389/fimmu.2025.1564333)
Supplement: Supplementary file 1 [file SupplementaryFile1.pdf]

Supplementary Material

Supplementary Table 1. Sex and age of all PBMC controls.

		**Cutaneous melanoma**	**Uveal melanoma**	**Healthy donors**
Sex	Females<br>Males<br>Total	3 (21%)<br>11 (79%)<br>14	7 (50%)<br>7 (50%)<br>14	8 (62%)<br>5 (38%)<br>13
Age	(Median + range)<br>Females<br>Males<br>Total	 73 years (71-79)<br>75 years (56-87)<br>75 years	 71 years (45-74)<br>65 years (55-80)<br>68 years	 74 years (71-75)<br>66 years (65-78)<br>73 years

Supplementary Table 2. Comparison of nucleotide sequences of CALPI-like CDR3 clonotypes and *TRDJ* primers conclude the former are an artifact and should not be included in the analysis.

**Name**	**Sequence (nt.)**
CALPI-like clonotype	TGTGCTCTTCCGATCT
TRDJ1	GTGACTGGAGTTCAGACGTGTGCTCTTCCGATCTCACAGTCACACGGGTTCCTT
TRDJ2	GTGACTGGAGTTCAGACGTGTGCTCTTCCGATCTCGATGAGTTGTGTTCCCTTTCCAA
TRDJ3	GTGACTGGAGTTCAGACGTGTGCTCTTCCGATCTAGTTTGATGCCAGTTCCGAAA
TRDJ4	GTGACTGGAGTTCAGACGTGTGCTCTTCCGATCTGTTGTACCTCCAGATAGGTTCCT

Supplementary Table 3. List of conjugated antibodies for flow cytometry.

Panel*	Antigen	Fluorophore	Clone	Manufacturer
Unstim, Stim	CD3	BV711	UCHT1	BD Biosciences (#563725)
Unstim	CD4	BUV737	OKT-4	BD Biosciences (#750977)
Unstim	CD8	PerCP-Cy5.5	RPA-T8	BD Biosciences (#560662)
Unstim, Stim	CD16	BUV395	3G8	BD Biosciences (#563785)
Unstim, Stim	CD161	PE	HP-3G10	BD Biosciences (#566843)
Unstim, Stim	NKG2D	PE-Cy7	1D11	BD Biosciences (#562365)
Unstim, Stim	TCRγ/δ	FITC	11F2	Miltenyi Biotec (#130-114-029)
Unstim, Stim	TCRVδ1	APC-Vio770	REA173	Miltenyi Biotec (#130-120-438)
Unstim, Stim	TCRVδ2	VioBlue	123R3	Miltenyi Biotec (#130-101-152)
Stim	Granzyme B	PerCP-Cy5.5	QA16A02	BioLegend (#372211)
Stim	IFN-γ	BUV737	4S.B3	BD Biosciences (#612845)
Stim	IL-17A	BV786	N49-653	BD Biosciences (#563745)

*Unstim = unstimulated panel, Stim = Stimulated panel

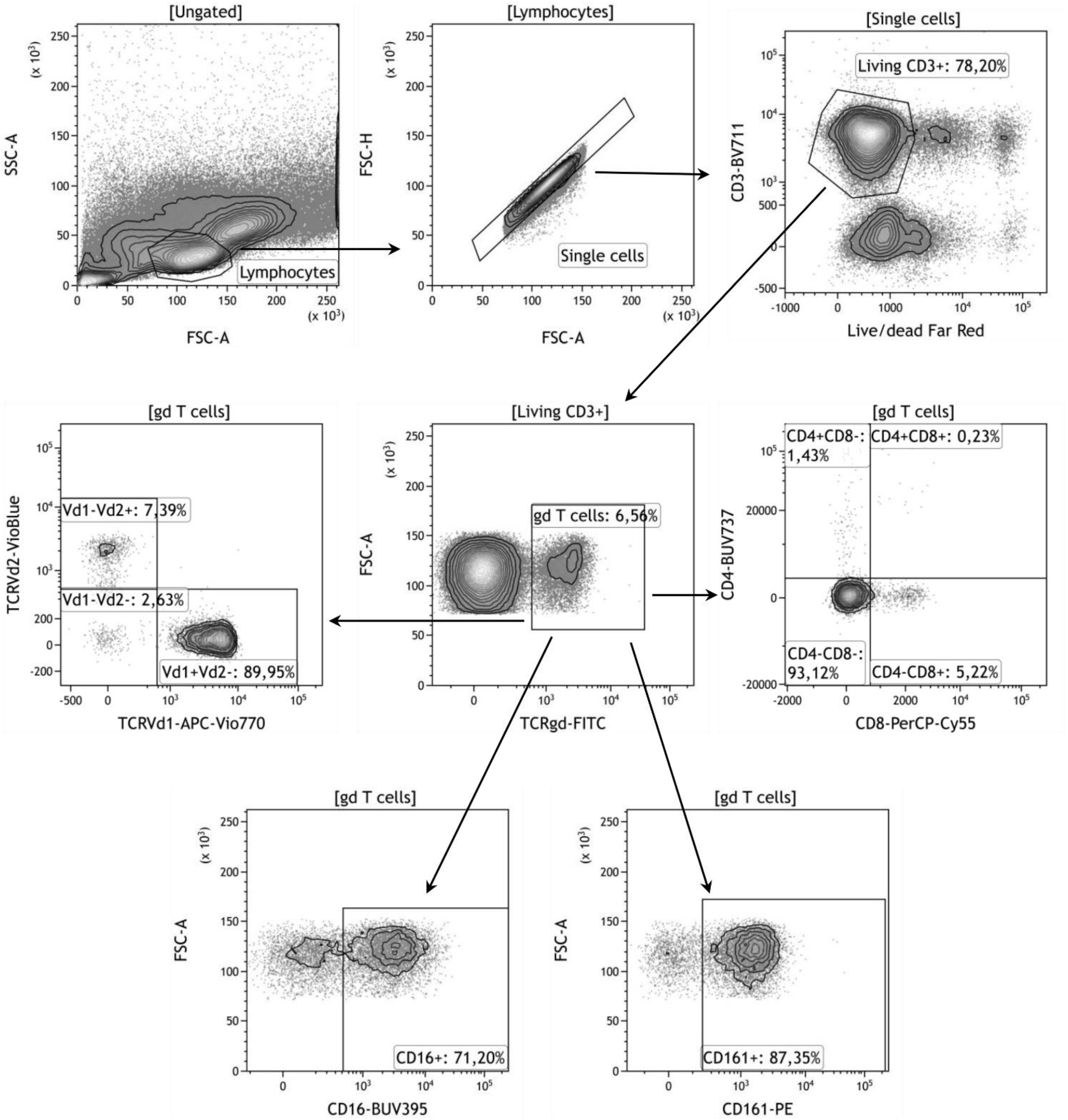

Supplementary Figure 1. Flow cytometry cell gating strategy for unstimulated samples

Flow cytometry plots showing the gating strategy for γδ T cells in peripheral blood from a representative unstimulated sample.

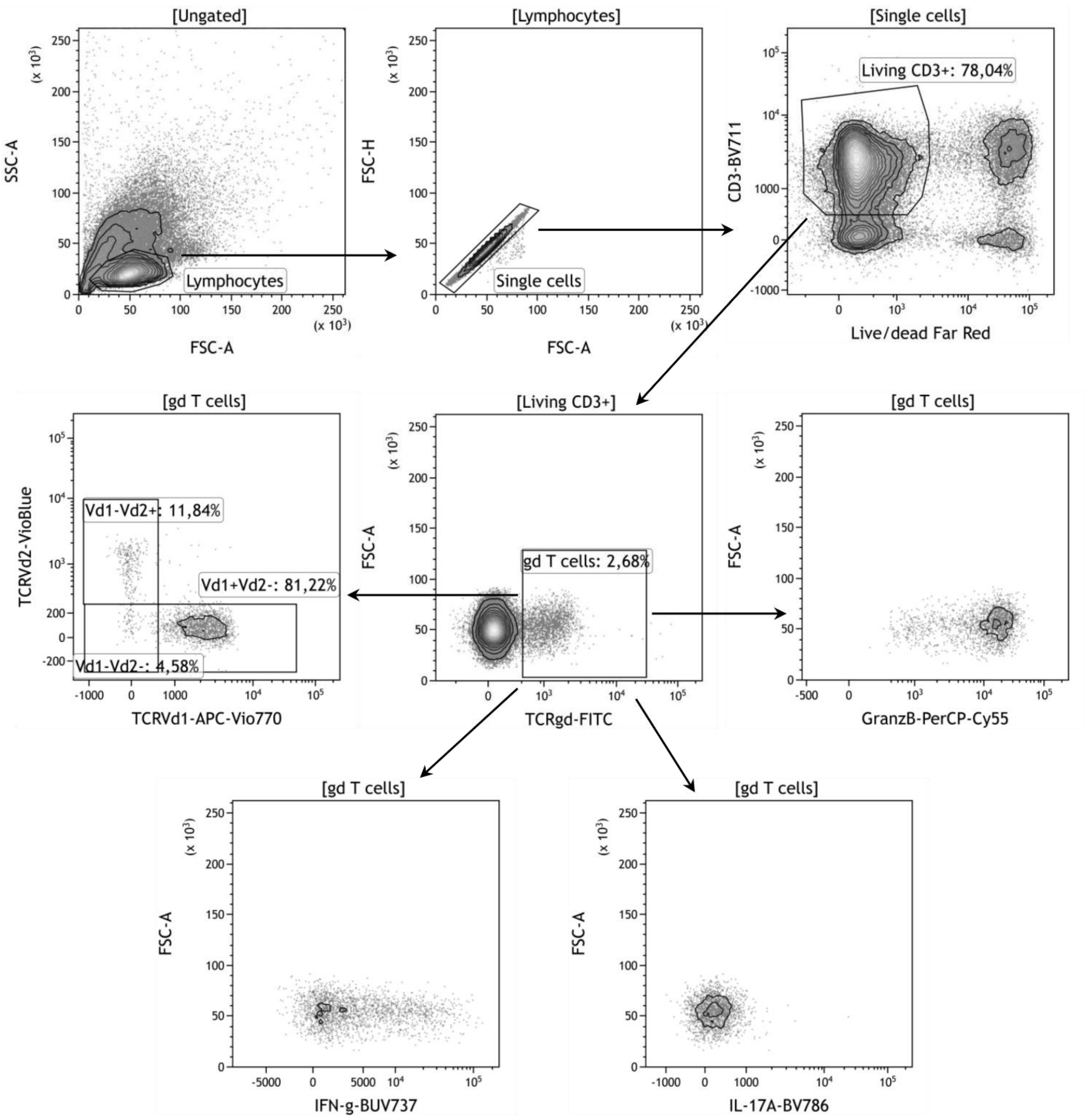

Supplementary Figure 2. Flow cytometry cell gating strategy for stimulated samples
Flow cytometry plots showing the gating strategy for γδ T cells in peripheral blood from a representative stimulated sample.

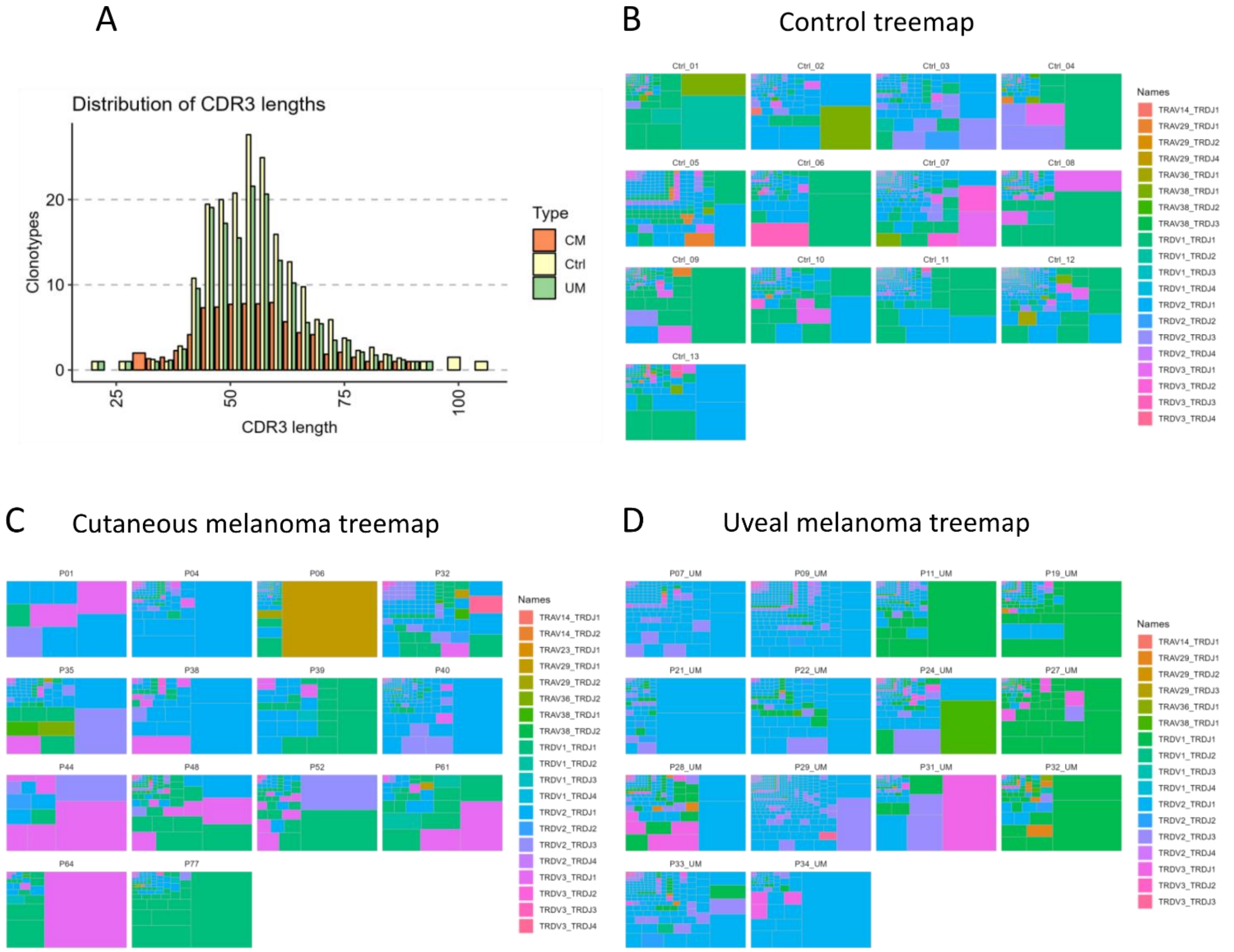

Supplementary Figure 3. Group clonality distribution
(A) Distribution of clonotypes by CDR3 nucleotide lengths within each group, following an approximate Gaussian curve, which means that not an individual clonotype stands out per group. And treemaps of clonotypes per sample within the **(B)** control, **(C)** cutaneous melanoma, and **(D)** uveal melanoma groups. Colors show the combination of *TRDV* and *TRDJ* genes per clonotype. Ctrl=Control, CM=Cutaneous melanoma, UM=Uveal melanoma.

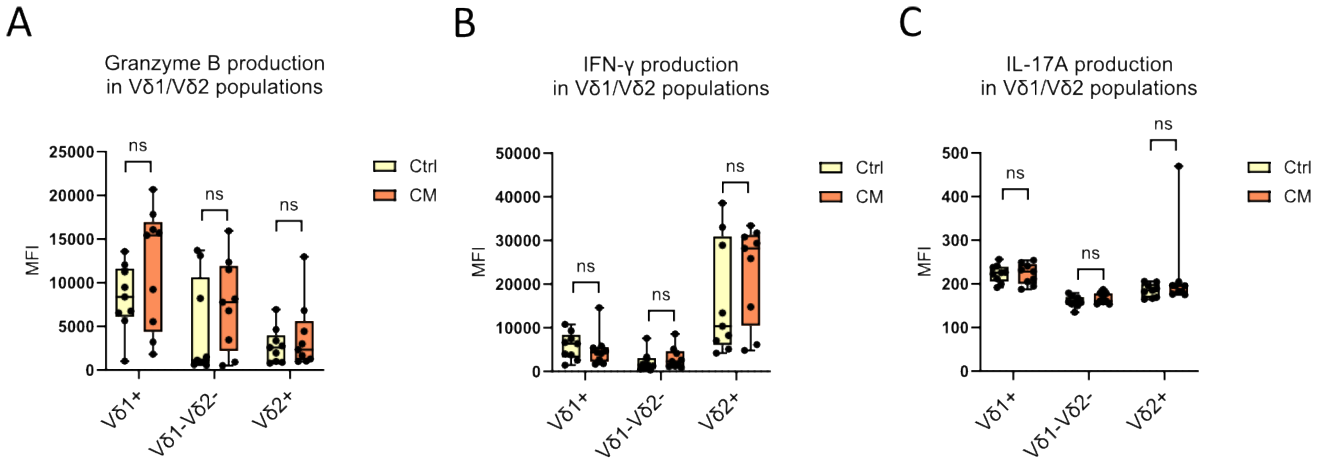

Supplementary Figure 4. Extended functionality studies

The intracellular expression of **(A)** granzyme B, **(B)** IFN-γ and **(C)** IL-17A, as measured by flow cytometry, in peripheral γδ T cells from cutaneous melanoma patients and healthy controls after 4 hour stimulation with PMA and ionomycin. n_{CM}=9, n_{Ctrl}=9, unpaired Mann-Whitney test, ns=not significant. MFI=Median fluorescence intensity, Ctrl=Control, CM=Cutaneous melanoma.
